# *Scolymus hispanicus* (Golden Thistle) Ameliorates Hepatic Steatosis and Metabolic Syndrome by Reducing Lipid Accumulation, Oxidative Stress, and Inflammation in Rats under Hyperfatty Diet

**DOI:** 10.1155/2021/5588382

**Published:** 2021-07-10

**Authors:** Sihem Berdja, Lynda Boudarene, Leila Smail, Samia Neggazi, Saliha Boumaza, Abdelhamid Sahraoui, El-mehdi Haffaf, Ghouti Kacimi, Souhila Aouichat Bouguerra

**Affiliations:** ^1^Laboratory of Cellular and Molecular Physiopathology, Faculty of Biological Sciences, University of Science and Technology Houari Boumediene, BP 32 DZ-16011 El Allia, Algiers, Algeria; ^2^Laboratory of Organic and Functionally Analysis, Faculty of Chemistry, USTHB, Algiers, Algeria; ^3^Laboratory of Nuclear Medicine of Central Hospital of Army, Algiers, Algeria; ^4^Laboratory of Biochemistry of Central Hospital of Army, Algiers, Algeria

## Abstract

**Background:**

Lipotoxicity is characterized by a metabolic disturbance leading to the development of nonalcoholic fatty liver disease (NAFLD). Some medicinal plant extracts exert hepatoprotective activity by modulating oxidative stress, inflammation, and metabolic disorders. *Scolymus hispanicus* or the golden thistle can be considered an important natural source of antioxidants. In traditional medicine, the consumption of this plant is recommended for diseases of the liver and intestines.

**Objective:**

In this study, we aimed to determine the effects of *Scolymus hispanicus* on a hyperfatty diet- (HFD-) induced metabolic disorders, oxidative stress, and inflammation.

**Materials and Methods:**

Our experiment focused on the administration of an HFD (40%) in *Rattus norvegicus* for 2 months and treatment with the aqueous extract of *Scolymus hispanicus* at a rate of 100 mg/kg during the last eight days of experimentation. In this context, several aspects were studied: the evaluation of blood biochemical parameters, liver function such as lipids and glycogen, markers of oxidative stress (TBARS, carbonyl proteins, advanced oxidation proteins, catalase, and SOD) and inflammation (NO and NFkB), morphological study of hepatocytes in primary culture, and histological study of the liver.

**Results:**

Lipotoxicity induced metabolic disorders, both serum and tissue. HFD induced an increase in the total lipids and a decrease in glycogen reserve and an alteration in the oxidant-antioxidant balance. HFD induced an increase in markers of liver damage, which resulted in NAFLD, confirmed by histological study and hepatocytes cell culture. *Scolymus* appears to have lipid-lowering, hypoglycemic, anti-inflammatory and antioxidant properties. It improved glucose tolerance and the condition of fatty liver disease.

**Conclusion:**

Golden thistle improves glucose tolerance and hyperlipidemia and ameliorates hepatic steatosis by reducing oxidative stress, inflammation, and lipid accumulation. Its incorporation into a dietary program or as an aliment supplement would prevent hepatic complications associated with an HFD.

## 1. Introduction

Overweight and obesity have become major global public health problems. Increasing consumption of more energy-dense, nutrient-poor foods with high levels of sugar and saturated fats and the increase in the availability of obesogenic ultraprocessed foods combined with reduced physical activity have increased obesity rates threefold or more since 1980 [1]. Overnutrition leads to excess calories, which induce the installation of obesity, indicating an imbalance in the energy balance, which occurs when the calories ingested are greater than those spent by the body. The intake will be higher and the storage lipids will therefore be increased. The increase in the storage of lipids and lipid derivatives leads to the expansion of adipose tissue (hyperplasia and hypertrophy) and the installation of lipotoxicity, which has harmful effects resulting in nonalcoholic fatty liver disease (NAFLD), which is associated with obesity [2].

NAFLD was recently redefined as metabolic-associated fatty liver disease (MAFLD) to reflect better the pathogenesis [2]. NAFLD is the most common chronic liver disease that affects around 25% of the population. NAFLD encompasses a broad spectrum of diseases that include simple fatty infiltration nonalcoholic steatohepatitis (NASH), which is defined as the presence of fat leading to inflammatory damage to hepatocytes, fibrosis, and finally cirrhosis. The importance of NAFLD lies in the possibility of its gradual progress to advanced fibrosis, cirrhosis, and hepatocellular carcinoma (HCC) [2, 3]. The overall prevalence of NAFLD is growing in parallel with the global epidemic of obesity [4]. The pathophysiology is complex and involves multiple concurrent mechanisms in the context of abnormal metabolic processes that arise mostly in individuals with risk factors. Comorbidities associated with NAFLD include obesity, type 2 diabetes (T2D), arterial hypertension, and dyslipidemia, as traits of metabolic syndrome (MetS) [3].

MetS is a clinical syndrome that includes obesity, dyslipidemia, arterial hypertension, and T2D [5]. NAFLD is strongly linked with all segments of MetS and it is in fact liver manifestation of MetS. Some authors have suggested that NAFLD could be defined as a fifth component of the MetS [5]. Both conditions were related to insulin resistance (IR), the main pathogenic factor underlying NAFLD and MetS. Abdominal fat overage is a fundamental determinant in NAFLD pathogenesis due to its association with IR and a possible source of free fatty acids (FFA) [3, 6]. Trunk fat was found to be indicative of elevated ALT, supporting the potential involvement of the metabolically active intra-abdominal fat in increased liver injury [2].

Obesity is associated with an increase in adipose tissue lipolysis, secretion of inflammatory, and fibrotic mediators, which can reach the liver. The accumulation of inflammatory/immune cells and the modification of the activities of these cells in the adipose tissue contributed to chronic low-grade inflammation during obesity [2, 3]. This sustained inflammation mediates IR and provides a contributing link between its development and NAFLD [2, 3]. The accumulation of hepatic diacylglycerol and the activation of inflammatory pathways are promoted. Diacylglycerols activate protein kinase *ε* and inhibit insulin signaling, leading to hepatic IR [2, 3]. The dysregulation of insulin-mediated control of hepatic production of glucose and lipids appears to be the main event in the development of NAFLD [3]. Normally, insulin impairs gluconeogenesis while promoting lipogenesis. There is a paradoxical situation in NAFLD, especially in the context of T2D. IR results in a reduced ability to inhibit gluconeogenesis but insulin-driven lipogenesis still occurs and is even enhanced [3].

Varieties of natural products have been proposed as a pharmacological treatment of MetS and T2D. *Scolymus hispanicus*, the golden thistle species, is food source and can be considered an important natural source of antioxidants. The golden thistle (*Scolymus hispanicus*), locally known as “Guernina” or “Thaghadiwth,” is one of the most popular plants in Algeria, Spain, and other Mediterranean countries [7]. In Algeria, we eat the petioles (“stems” of the leaf, or more exactly the main vein) cooked in the broth (red sauce with meat) that accompanies couscous.

*Scolymus hispanicus* has been linked to many medicinal properties such as diuretic, depurative, digestive, choleretic, and lithiuretic properties [7]. Moreover, in traditional medicine, consuming this plant in the green or cooked state is recommended for liver and intestines diseases [8]. The flaky stems are used for digestive tract care, bronchitis, and cold and have emmenagogic and antidiarrhoeal properties [9]. The roots in decoction are recommended as an antidiabetic. Consumption of the ribs (main veins) of this plant fresh or cooked is recommended for liver and intestinal diseases [8]. Other uses in the traditional medicine of golden thistle have been reported, such as in Malta fever and eye infection [10]; it can also be used as an appetizer and as a hemostatic agent [11]. The antioxidant activity of *Scolymus* has been reported [10, 12].

Phytochemical analysis has demonstrated that the plant contains many biologically active compounds and a high content of *α*-tocopherol and identified 3 flavonoids (catechin, rutin, and tannic acid) and 13 phenolic acids, such as gallic acid, pyrogallol, chlorogenic acid, *p*-hydroxybenzoic acid, vanillic acid, caffeic acid, syringic acid, *p*-coumaric acid, ferulic acid, sinapic acid, salicylic acid, and rosmarinic acid resveratrol [13].

In this context, the present study aims to evaluate the effect of the aqueous extract of *Scolymus hispanicus* on HFD-related metabolic disorders, steatosis hepatic, inflammation, and stress markers.

## 2. Materials and Methods

### 2.1. Preparation of Aqueous Extract from *Scolymus hispanicus*

The aerial part of *Scolymus hispanicus* or the golden thistle was harvested in Algiers in February 2019. The voucher specimen (INA/P/No 54) has been preserved in the herbarium of the Botany Department, National Institute of Agronomy (INA), Algiers, Algeria. The stems and leaves of *Scolymus* were washed and separated from the roots, cut into small slices, dried, then added to 1000 mL of water, and left to boil for 50 min on a thermostated stirrer. After the cooling, the extract was filtered through muslin. The filtrate was centrifuged at 1500 rpm for 5 min and a second time at 2000 rpm for 10 min to obtain a homogeneous liquid. After the centrifugation, all samples were filtered through filter paper (Whatman with a pore size of 11 *μ*m). The collected aqueous extract was then lyophilized (Cryodos 80, −75°C, 5 m^3^/h) to find an extract yield of 4.3%. The extract was stored in sealed glass vials at ± 4°C before being tested and analyzed.

### 2.2. Preparation of the Hyperfatty Diet

The hyperfatty diet at 40% was prepared in the cellular and molecular physiopathology team/BPO Laboratory/USTHB. According to the recommended nutritional intake, fats should not exceed 30% of the total daily energy intake to avoid unhealthy weight gain. In our study, we used a rate of 40% of lipids to confirm the installation of obesity with metabolic dysfunctions in the rats. The hyperfatty diet is based on cooked sheep fat; the cooking increases saturated fatty acids. The lipid intake in these rats is represented by 40 g of cooked sheep fat equivalent of 360 kcal; this fat is added to 60 g of the standard laboratory food equivalent of 186 kcal to constitute 100 g of food equivalent of 546 kcal. A daily diet of 20 g of hyperfatty food provides 109.2 kcal/day.

### 2.3. Animals

This study was carried out on 28 female rats of the *Rattus norvegicus* with average weights of 111.33 ± 27.66 g, which were reared at the animal facility of the Faculty of Biological Sciences, USTHB, with controlled temperature (22 ± 1°C), lighting (12-hour dark/light cycle), and free access to food and water.

The animals were divided into 4 groups:

1. Control batch: seven control rats subjected to a standard laboratory diet for 2 months of experimentation. The feed was provided by the National Animal Feed Office; the calories intake contained in 20 g of food is 62 calories.
2. Control batch treated with the aqueous extract of *Scolymus hispanicus* at a rate of 100 mg/kg of body weight/day during the last eight days of experimentation by intraperitoneal injection (7 animals).
3. Batch subjected to a hyperfatty diet (HFD) at 40% for two months with a daily intake of 20 g per rat. The calorie intake contained in 20 g of food was 109.2 calories.
4. Batch subjected to an HFD and treated with the aqueous extract of *Scolymus hispanicus* at a rate of 100 mg/kg of body weight/day during the last eight days of experimentation by intraperitoneal injection while maintaining the hyperfatty diet (7 animals).

### 2.4. Methods

#### 2.4.1. Chemical Study

*(1) Total Phenolic Content*. The content of total polyphenols in the aqueous extract of *Scolymus hispanicus* was determined using the Folin–Ciocalteu reagent according to the method of Singleton et al., using gallic acid as a reference [14]. An aliquot of the aqueous (0.2 mL) extract contains 1000 *μ*g of *Scolymus* mixed with 46 mL of distilled water and 1 mL of Folin–Ciocalteu reagent in a volumetric flask. The mixture was incubated for 3 min in the dark. After that, 3 mL of sodium carbonate solution (7.5%) was added to the mixture. After 2 hours of incubation in the dark, the absorbance was measured at 740 nm in a spectrophotometer (Shimadzu 1800, Mulgrave, Victoria, Australia). The total phenolic content was evaluated from a standard calibration curve of gallic acid, and the results were expressed as micrograms of gallic acid (GA) equivalents (*E*) per milligram of extract (*µ*g GAE/mg).

*(2) Determination of Total Flavonoids.* The total flavonoids were determined according to the modified method described by Lebreton et al. using quercetin as a reference [15]. Four milliliters (4 mL) of dilution solution was mixed with 4 mL of aluminum trichloride solution (2% in methanol). After 15 min of incubation, the absorbance was measured at 415 nm. Quercetin (*Q*) was used as a reference compound to produce the standard curve. The results were expressed as *μ*g QE/mg.

*(3) Antioxidant Activity: Scavenging Effect on DPPH Radical*. The 2,2-diphenyl-1-picrylhydrazyl (DPPH) free radical scavenging assay was carried out as described by Brand-Williams et al. [16]. It is based on the degradation of the DPPH radical dissolved in an 80% methanol/water mixture. An antioxidant will have the ability to donate an electron to the synthetic radical DPPH (purple coloration) to reduce it to nonradical DPPH (yellow-green coloration). The aqueous extract was dissolved in methanol. A sample of 25 *μ*L of each concentration (100, 200, 400, 600, 800, and 1000 *μ*g/mL) was added to the DPPH methanol solution (60 *μ*M, 975 *μ*L). After 30 min of incubation at 25°C, the absorbance at 517 nm was measured by UV spectrophotometer (Jasco, V-530). Ascorbic acid and *α*-tocopherol were used as compounds reference. The radical scavenging activity was then calculated using the following equation: % of radical scavenging activity = ((Abs control−Abs sample)/Abs control) × 100, where Abs control is the absorption of the blank sample and Abs sample is the absorbance of the tested extract.

#### 2.4.2. Biological Study

*(1) Analytical Techniques*. The animals were bled from the retroorbital venous plexus; this technique eliminates using anesthetic agents affecting measurements of biochemical parameters. Blood, which was collected in dried tubes, was centrifuged at 3000 rpm for 10 min and the sera were stored at −20°C. Blood glucose, triglycerides, cholesterol, and transaminase were measured by enzymatic colorimetric method using a test kit of Biosystem. Blood insulin was determined by radioimmunoassay using a CIS test kit (ORIS INDUS). The evaluation of the redox status was performed in the sera and erythrocytes by assaying the thiobarbituric acid reactive substances (TBARs) and catalase.

*(2) Oral Glucose Tolerance Test (OGGT).* The oral glucose tolerance test (OGTT) measures the clearance of glucose from the body after its absorption from the intestinal tract. All rats were weighed one day before the test for the calculation of the glucose solution to be administered. The glucose solution (40%) was administered by intraperitoneal injection. Rats received 2 mg of glucose/g of body weight [17, 18].

The rats were fasted for a period of 14 to 16 hours with free access to water. A blood sample was taken from a small incision in the tail using a scalpel to measure basal blood glucose level (=time point 0) with the glucometer *vital check* [17, 18]. Once basal glucose concentrations were measured in all rats, the glucose solution was given to each animal by intraperitoneal injection. The timer was immediately started after the first administration of glucose to all rats. After 30 min, the blood glucose was measured using a glucometer of each rat in the same order as they were injected. This operation was repeated in 60, 90, and 120 min after glucose administration [19, 20].

*(3) Organs Harvesting*. At the end of the experiment, animals were sacrificed after anesthesia by intraperitoneal injection of urethane. The liver removed was divided into five fragments, and each fragment was weighed. They were intended for different assays, including total lipids where the fragment is immersed directly in Folch solution. Another fragment was bound in paraformaldehyde at 10%, and the other three fragments were frozen directly in liquid nitrogen to evaluate redox status, inflammatory markers, and hepatic glycogen. Two animals from each batch were kept for the initiation of hepatocyte cell culture.

*(4) Histology of the Liver*. After fixation in paraformaldehyde at 10% for 24 h, the specimens of liver were dehydrated and embedded in paraffin and cut at 5 *μ*m. The sections were stained with Masson's trichrome [21].

*(5) Hepatic Glycogen.* The principle of the method consists in hydrolyzing the glycogen extracted from the liver of rats into glucose with an acid and determining the amount of the formed glucose using the Folin and Wu method [22]. Concentrations were deduced from a standard curve prepared with standard glucose solution and the amount of glycogen was expressed per 100 g of liver.

*(6) Total Lipids.* The extraction was carried out according to the method of Folch et al. [23]. The lipids were extracted using chloroform/methanol (2 : 1 *v*/*v*). The total lipids were estimated in mg/100 g of liver.

*(7) Oxidant and Antioxidant Activity*.

- *Catalase Activity Assay*. The enzymatic activity of catalase was determined using the method of Claiborne [24]. The principle was based on the disappearance of H_2_O_2_ in the presence of the enzyme source at 25°C. Catalase was evaluated in sera, erythrocytes, and liver of all animal groups. Absorbance was estimated at 240 nm in two time points, *t*0 and after two min. Erythrocytes and liver were lysed, before all assays, in a lysis buffer [25].
- *Superoxide Dismutase (SOD) Activity Assay*. The evaluation of the SOD activity was performed according to the method of Giannopolitis and Ries [26].
- *Thiobarbituric Acid Reactive Substances Assay (TBARs)*. After the reaction with thiobarbituric acid (TBA) (Sigma) [27], the TBARs were measured in sera, erythrocytes, and liver. The MDA contained in the supernatant in the presence of 10% trichloroacetic acid reacted with TBA and caused the formation of a red complex estimated at 532 nm.
- *Protein Carbonyl Assay*. Protein carbonyls (PC) were measured in the liver of all animal groups according to the procedure described by Reznick and Packer [28] using dinitrophenylhydrazine (DNPH) reagent and spectrophotometric method. The absorbance was measured at 370 nm. The results were expressed as nanomoles of carbonyl groups per milligram of protein using a molar extinction coefficient of 22 000M^−1^ cm^−1^.
- *Advanced Protein Oxidation Products Assay.* The determination of advanced protein oxidation products (AOPP) levels was performed in the liver by modifying the Witko-Sarsat method [29]. The absorbance of the reaction mixture was immediately estimated at 340 nm. AOPP concentrations were expressed as micromoles/L of chloramine-T equivalents [30].

**(** *8) Measurement of Inflammation Markers*.

- *Nuclear Factor-Kappa B (NFκB).* The assessment was determined by immunoenzymatic assay. Invitrogen ELISA kits were used for measuring the levels of the NF-kB p65 in the liver of all groups. The estimation was made by Elisa reader at 450 nm (BioTek Instruments).
- *Nitrogen Monoxide Assay (NO).* The determination of nitrite and nitrate was evaluated from supernatants of the liver of different groups. The nitrite bearing in all samples, which were deproteinized and regenerated, was quantified after addition of Griess reagent (0.1% N-(1naphthyl) ethylenediamine dihydrochloride, 1% sulfanilamide, and 5% phosphoric acid). Absorbance was measured at 543 nm [31].

*(9) Perfusion and Isolation of Hepatocytes*. This technique was carried out using the modified method of Severgnini et al. and Edwards et al. [32, 33]. All steps were performed under sterile conditions.

After anesthesia of the animals by intraperitoneal injection with urethane, insert the cannula in the portal vein and start the perfusion using a peristaltic pump containing phosphate-buffered saline (PBS) at pH 7.2. As soon as the infusion starts, immediately cut the hepatic vein to allow perfusate to run as waste.

The flow was maintained at 5 mL/min for 15 to 20 min thanks to the peristaltic pump to remove the blood completely in each lobe. A second solution at pH 7.4 containing trypsin replaces PBS for tissue digestion. At this stage, the hepatic tissue was rapidly disaggregated.

The liver was collected with a curved spatula and was transported in a sterile Petri dish containing DMEM + fetal calf serum (FCS), where we proceeded with the disruption of the tissue proceeded using a scalpel. This step should be fast in order to avoid damage to hepatocytes.

The medium containing the cells was recovered, followed by centrifugation at 600 rpm for 5 min at room temperature. The supernatant was subsequently removed and the cells were suspended a second time in 30 mL of Percoll cushion at 37.5% for recovering viable cells. Another centrifugation was effectuated for 3 minutes at 1000 rpm at room temperature.

- *Hepatocyte Culture and Microscopy.* The viable cells recovered were suspended again in 2 mL of DMEM; the hepatocytes are observed with an inverted microscope after staining with trypan blue. The cells were distributed in flasks, which were adjusted to 5 mL of DMEM supplemented with FCS, L-glutamine, and antibiotics, and they are incubated in a CO_2_ incubator (Memmert) (5% CO_2_, 95% air) for the start of the primary culture. After 48 h of incubation, we noted the confluence of the cells. Trypsinization was necessary to perform the first passage [32, 33].

#### 2.4.3. Statistical Analysis

Data were analyzed with ANOVA using STATISTICA version 6 and completed with HSD Tukey's test. The results were expressed as the mean ± standard deviation. The differences at ^*∗*^*p* < 0.05 were considered to be statistically significant.

## 3. Results

### 3.1. Phytochemical Study of *Scolymus hispanicus*

The aqueous extract of *Scolymus hispanicus* showed a high content of total polyphenols and flavonoids (Table 1). The antioxidant activity of the aqueous extract of *Scolymus hispanicus* was evaluated using the DPPH free radical scavenging test. Our extract showed a very important antifree radical activity with an IC50 value of 0.0038 *µ*g/ml, which was extremely higher than the reference values BHA and BHT (21.18 ± 0.12 *µ*g/mL and 12.66 ± 0.18 *µ*g/mL, respectively) (Table 1).

### *3.2. Scolymus hispanicus* Improved the Metabolic Disorder and Reduced Body Weight Gain after Eight Weeks of Hyperfatty Diet

As illustrated in Table 2, baseline body weight and biochemical parameters were similar between the four study groups of *Rattus norvegicus*. After eight weeks of hyperfatty diet, body weight was higher than that in normal diet-fed animals (Table 2). In terms of biochemical parameters, the administration of hyperfatty diet induced an increase in plasma levels of glycemia, insulinemia, total lipids, including triglycerides, and total cholesterol, characterizing the metabolic syndrome known as insulin resistance (Table 2). The evaluation of transaminase (AST and ALT) was found to be significantly increased in terms of experimentation in an animal group subjected to a hyperfatty diet compared to the control (Table 2).

The treatment with *Scolymus hispanicus* at a rate of 100 mg/kg of body weight/day during the last eight days of experimentation by intraperitoneal injection corrected the metabolic disorders by reducing glycemia, insulinemia, triglyceridemia, and cholesterolemia and amelioration of glucose tolerance compared to animals submitted to hyperfatty diet (HFD) (Table 2) and protected the animals against hepatic steatosis complication by decreasing transaminase levels.

### 3.3. *Scolymus* Improved Glucose Tolerance after Eight Weeks of HFD Feeding

**Figure 1:**
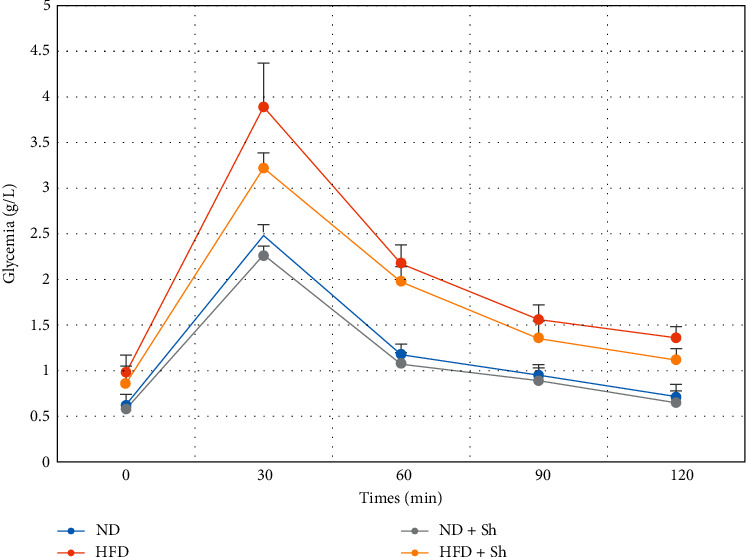
Oral glucose tolerance test in control and experimental groups. Effect of *Scolymus hispanicus*. Data were expressed as mean ± standard deviation (SD) (*n* = 7). ND: normal diet; HFD: hyperfatty diet (40%) for eight weeks; ND + Sh: normal diet + *Scolymus hispanicus* extract (100 mg/kg of body weight/day during the last 8 days of experimentation); HFD + Sm: hyperfatty diet treated with aqueous extract of *S. hispanicus*. *p* > 0.05 was considered statistically not significant; *p* < 0.05 was considered statistically different.

Experimental induction of hyperglycemia by an intraperitoneal injection of glucose induced an increase in blood glucose, revealing impaired glucose tolerance in the HFD animal group (Figure 1). After the administration of *Scolymus,* blood glucose levels were significantly lower at 30 and 60 min after glucose load in HFD rats treated with *Scolymus* than that in the HFD group (Figure 1).

### 3.4. Golden Thistle Ameliorates the Oxidant-Antioxidant Balance in Blood

The evaluation of redox statues in the blood (sera and erythrocytes) indicated a significant increase in lipid peroxidation (TBARs) with a decrease in the catalase activity (Table 3). The treatment with *Scolymus* decreases significantly the rate of TBARs and increases the catalase activity (Table 3).

### 3.5. *Scolymus hispanicus* Increased Hepatic Glycogen Storage and Reduced the Hepatic Lipid Accumulation after Hyperfatty Diet

Hepatic glycogen was decreased in the animals subjected to the hyperfatty diet; thus, the hepatic storage of glycogen was altered. On the other hand, we noted a significant increase in the quantity of total hepatic lipids, characterizing the installation of hepatic steatosis (Table 4).

As illustrated in Table 4, the administration of *Scolymus hispanicus* to animals subjected to HFD induced a significant increase in the liver glycogen content accompanied by a decrease in total liver lipid. *Scolymus* ameliorates the storage of hepatic glycogen and reduces lipid accumulation.

### 3.6. *Scolymus hispanicus* Attenuated Oxidative Stress in Liver

The evaluation of liver catalase activity showed a decrease in the HFD group compared to control, accompanied by an increase in liver SOD activity. The evaluation of stress markers such as TBARs AOPP protein carbonyl showed a significant increase compared to controls (Table 5).

As shown in Table 5, *Scolymus hispanicus-*treated rats showed a decreased oxidative stress by increasing catalase and SOD enzyme activity and reducing lipid peroxidation (TBARs) and protein oxidation (PC, AOPP).

### 3.7. *Scolymus hispanicus* Attenuated Hepatic Inflammation Induced by Hyperfatty Diet

The evaluation of two markers of inflammation, such as total nitrite and NF*κ*B, showed a significant increase compared to the control group. The treatment with *Scolymus* reduces the inflammation by decreasing the levels of total nitrite and NF*κ*B (Table 6).

### 3.8. *Scolymus* Alleviates Fatty Liver Disease after Hyperfatty Diet

**Figure 2:**
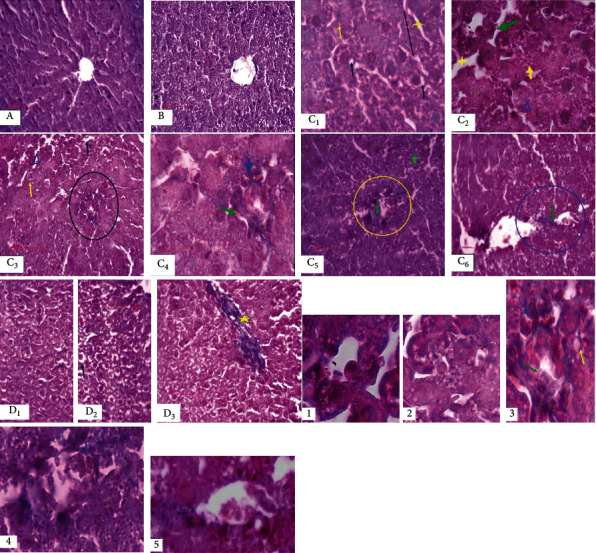
The effect of the *Scolymus hispanicus* extract on hepatic histological changes of the rats after Masson's trichrome: (A) normal diet group; (B) normal diet + Sh group; (C) hyperfatty diet group (40% during for 8 weeks); (D) hyperfatty diet + *S. hispanicus* at 100 mg/kg/day for 8 days. (1, 2) extension of C2 figure showing, respectively, the infiltration of monocytes and the chemotaxis phenomenon, hepatic steatosis marked by the accumulation of lipid droplets. Picture 3: enlargement of the zone of inflammation highlighting red blood cells and adhesion inflammatory cells. (4) Enlargement of C5 figure showing a partial thrombus with initiation of chemotaxis represented by a green arrow. (5) Enlargement of C6 figure showing the inflammatory process marked by the adhesion of monocytes to the vascular walls (initiation of chemotaxis) represented by a green arrow. Yellow arrow: hepatic lipid droplets; yellow star: widening of the sinusoidal space; blue triangle: disorganization of the cellular structure; green arrow: infiltration of inflammatory cells; black circle: inflammatory hemorrhagic focus marked by the presence of red blood cells (light blue arrow) and the inflammatory cells (monocyte and lymphocyte) (green arrow); yellow circle: partial thrombus of the hepatic portal space; green star: perivascular fibrosis; yellow stars: persistence of fibrosis.

As illustrated in Figure 2, Masson's trichrome staining showed a normal histological liver architecture formed of hepatic lobules. Briefly, each lobule was made up of radiating plates. Strands of cells form a network around a central vein with myofibrils and muscle bundles in liver sections from the normal diet-fed animals and NFD treated with *Scolymus*.

After eight weeks of hyperfatty diet, we recorded structural alterations in the tissue compared to the controls (ND and ND + Sh); these alterations were marked mainly by lipid deposit accumulation within the hepatocyte under the form of lipid droplets. The latter are present in the form of lipid micro- and macrovesicles, marking the installation of hepatic steatosis. We also observed the infiltration of inflammatory cells associated with interstitial fibrosis marking the onset of inflammation. In addition, we also noted the hypertrophy of the hepatic cells, a disorganization of the cellular architecture, and the widening of the sinusoidal spaces (Figure 2).

Treatment with *Scolymus hispanicus* induced attenuation of hepatic steatosis with a decrease in lipid micro- and macrovesicles, an improvement in cell structure, and a decrease in hypertrophy compared to the HFD group. We also noted the persistence of fibrosis and the absence of inflammatory infiltration in the HFD + Sh group compared to the HFD group (Figure 2).

### 3.9. Morphological Study of Hepatic Cells in Primary Culture: Effect of *Scolymus hispanicus* after Hyperfatty Diet

**Figure 3:**
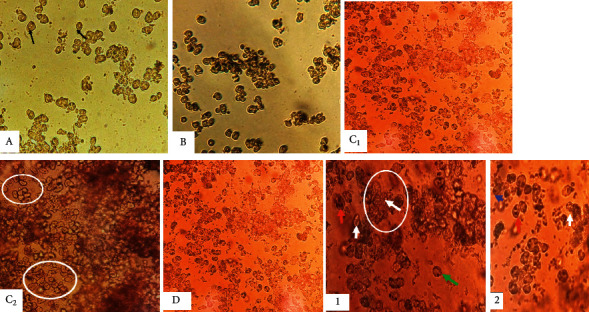
The effect of the *Scolymus hispanicus* extract on hepatic cell changes in primary culture. (A) Cells from animals subjected to the normal diet group; (B) cells from animals subjected to the normal diet + Sh group; (C1, C2) cells from animals subjected to the hyperfatty diet group (40% during for 8 weeks); (D) cells from animals subjected to the hyper atty diet + *S. hispanicus* at 100 mg/kg/day for 8 days. (1) Enlargement of C2 figure showing liver cells from rats subjected to HFD with lipid droplets and some cells with eccentric nuclei. (2) Enlargement of C2 figure showing liver cells from rats subjected to HFD treated with *Scolymus* with clear mononuclear and binuclear cells. Blue arrow: small clear mononuclear liver cells; red arrow: small clear binuclear liver cells; white circle and white arrow: lipid droplets; green arrow: cells with eccentric nuclei.

Observation of control hepatocytes (ND, ND + Sh) in primary culture revealed cells of small size, clear, round or oval, mononuclear or binucleate (Figure 3).

Observation of *Rattus norvegicus* liver cells subjected to HFD in primary culture showed cells larger in size compared to controls, irregular in shape, mononuclear or binucleate, with lipid vesicles. Some cells have eccentric nuclei (Figure 3).

Treated with aqueous extract of *Scolymus hispanicus* improved the cellular appearance of hepatocytes subjected to HFD in primary culture marked by the reduction of lipid droplets (Figure 3).

## 4. Discussion

The present results showed that eight weeks of hyperfatty diet leads to a metabolic syndrome associated with several structural changes in the liver characterized by lipid accumulation, inflammation, and oxidative stress in *Rattus norvegicus*. Moreover, the extract of *Scolymus hispanicus* seems to have lipid-lowering, anti-inflammatory, and antioxidant properties in *Rattus norvegicus* subjected to HFD. It improves the condition of fatty liver disease. The presence of bioactive molecules in the extract of *Scolymus hispanicus* could give this interesting antioxidant and hepatoprotective potential.

The aqueous extract of *Scolymus hispanicus* showed a high content of total polyphenols and flavonoids compared to other plants [25]. Our extract showed a very important antifree radical activity compared to the reference BHA and BHT. The content of the total polyphenols and flavonoids and the antioxidant activity of our extract are very high compared to the results found by Morales et al. [10] on *Scolymus hispanicus* in Spain. This variability is linked to the nature of the soil and to the geographical location.

In our study, the 40% hyperfatty diet (HDF) induced the onset of obesity characterized by the increased body weight. This result is in agreement with the study Hamalt et al. [34] and Harrat et al. [35], who showed that a 40% hyperfatty diet induced an increase in the body weight in Wistar rats. It is possible that the mechanisms regulating appetite respond more slowly to fat than to protein and carbohydrates. Thus, dietary fat has little effect on satiety and periodic exposure to hyperfatty diet and the increase in food density portion size increased availability and promoted obesity [36, 37].

Treatment with the extract of the plant *Scolymus hispanicus* induced a decrease, however not significant, in the body weight. This is probably due to the duration of the treatment. A period of 8 days would not, therefore, be sufficient to have conclusive results. Our results were in agreement with the work of Sayin et al. [38], which showed that the treatment of animals subjected to a hyperfatty diet with ethanolic extract of *Silybum marianum*, belonging to the same family as *Scolymus*, for 4 months had no effect on weight change, while the 11-month treatment reduced the body mass.

### 4.1. Metabolism Disorder

The hyper fatty diet caused metabolism disorders in rats marked by the hyperglycemia associated with hypertriglyceridemia, hypercholesterolemia, and hyperinsulinemia and impaired glucose tolerance at the end of the experiment. These results characterized the installation of metabolic syndrome. Our work agrees with that of Lee et al. [39]; this can be explained on the one hand by the preferential oxidation of fatty acids, which leads to a defect in the use of glucose via the inhibition of the key enzymatic activities of glycolysis, which will block the entry of glucose into the cells. Oxidation of fatty acids also provides key cofactors of gluconeogenesis, which increases the production of glucose by the liver and leads to an increase in circulating glucose. On the other hand, fatty acids interfere with insulin signaling pathways [40, 41]. Our work is in agreement with the work of Murakami et al. [42]; the hyperlipidemia observed can be explained by the high lipid content in the diet since the long-term administration of a hyperfatty diet causes both plasma and tissue disorders [34, 43].

Treatment with *Scolymus hispanicus* extract led to a decrease in the blood sugar level, decrease in insulin level, and improvement in glucose tolerance. Our results agree with the work of Feng et al. [44]. The polyphenols present in the plants of the Asteraceae family improve the endocrine function of the pancreas and insulin sensitivity [38] and promote functional recovery of the insulin receptor substrate 1 [44]. Marmouzi et al. reported the relevant antioxidant effect of *Scolymus hispanicus* functional parts and their antidiabetic activities via *α*-glucosidase and *α*-amylase inhibition [13].

*Scolymus* induced a decrease in cholesterolemia and triglyceridemia, which is consistent with the work of Sayin et al. [38]. This is explained by the decrease in the transcription factor SREBP-1c under the effect of *Silybum marianum*. The latter had the regulatory role of the genes involved in the synthesis of fatty acids and in the metabolism of hepatic triglycerides and cause also decreased expression of the fatty acid synthase (FAS) gene and attenuation of acetyl-CoA carboxylase (ACC) activity. The latter two play a role in inhibiting de novo lipogenesis [38, 45].

### 4.2. Lipotoxicity and Hepatic Function

The hyperfatty diet induced an increase in glutamic oxaloacetic transaminases and glutamic-pyruvic transaminases in the rats subjected to HFD, which confirms the work of Maximos et al. [46]. They are considered to be biomarkers of liver dysfunction and liver damage caused by hyperfatty diet. In obesity, the high amounts of toxic metabolites lead to the depletion of hepatic glutathione due to the increase of free radicals. These oxidants cause necrosis in liver cells, inducing an increase in the concentration of aminotransferases and their release into the serum [47].

Treatment with *Scolymus* extract resulted in a decrease in the levels of transaminases compared to the HFD group, which is in agreement with the work of Zhang et al. [48]. The polyphenols present in plants of the Asteraceae family induce an improvement in hepatic steatosis and inflammation thanks to their hepatoprotective properties and by restoring hepatic markers [38].

Our study showed an increase in total hepatic lipids in animals on a 40% hyperfatty diet, which results in metabolic dysfunction, leading to the accumulation of lipids in the hepatocytes. This dysfunction affects all stages of lipid metabolism, which is primarily an excessive uptake of free fatty acids produced by lipolysis in adipose tissue, an accumulation of triglycerides, an increase in the hepatic lipogenesis concomitant with a decrease in *ß*-oxidation, and finally a decrease in the secretion of VLDL [49].

Golden thistle induces a decrease in the quantity of lipids and triglycerides stored in the liver. Our results agree with those carried out by Aoun et al. [50]. A number of studies have suggested that the polyphenols contained in milk thistle promote the use of stored fatty acid as an energy source, which would primarily explain this decrease [51]. Secondly, the active compounds of milk thistle lead to the stimulation of genes involved in the inhibition of hepatic lipogenesis and the regulation of lipoprotein metabolism by increasing the synthesis of HDL and decreasing the synthesis of VLDL. Flavonoids treatment decreases the development of nonalcoholic fatty liver disease [52].

Our results showed a decrease in liver glycogen in animals subjected to HFD, which does not agree with the studies by Auberval et al. [43]. According to Magnan [53], diets rich in lipids cause the intracellular increase in free fatty acids and induce, on the one hand, the direct inhibition of the main enzymes of glucose metabolism.

An increase in hepatic glycogen was observed in animals treated with the aqueous extract of *Scolymus*, which is consistent with the data reported by Guigas et al. [54]. This indicates the potent antihyperglycemic role of *Scolymus*. A decrease in gluconeogenesis and glycogenolysis associated with reduced hydrolysis of glucose-6-phosphate plays a key role in the regulation of hepatic glycogen metabolism [54].

The hyperfatty diet caused macrovesicular steatosis, which results in ectopic accumulation of triglycerides in the cytoplasm of hepatocytes and inflammatory infiltration of liver tissue by polymorphonuclear cells and mononuclear cells, which are mainly lymphocytes that led to the evolution to steatohepatitis then to fibrosis, consistent with the work of Savard et al. [55]. This is explained, on the one hand, by the metabolic disturbance that induced the increase in the influx of free fatty acids and the activation of de novo lipogenesis and, on the other hand, by oxidative stress that induced a decrease in hepatic ATP production and the production of proinflammatory cytokines, which trigger inflammatory necrosis leading to the progression of steatohepatitis [56].

Hepatic steatosis was alleviated after treatment with golden thistle, which is in agreement with the work of Ni and Wang [57]; this can be explained by the presence of polyphenols, which reduce the accumulation of triglycerides and improve the severity of fatty liver disease [58]. We have also noted the persistence of fibrosis. This may be related to the short duration of treatment and the dose administered.

The morphological study of hepatic cells in primary culture strengthens and approves the liver histological results obtained following treatment with *Scolymus*.

### 4.3. Lipotoxicity, Redox Status, and Inflammation

Our results concerning the markers of the antioxidant status showed that there was a decrease in the antioxidant capacity of catalase in the serum, the erythrocyte, and the liver in the rats subjected to a hyperfatty diet of 40%. These results were in agreement with the work of Furukawa et al. [59]. An increase in the flow of fatty acids to the liver, an excess of hepatic synthesis, and an imbalance in the diet cause a decrease in catalase. CAT is an enzyme, which catalyzes the disproportionation of hydrogen peroxide into oxygen and water. The initial excess of fatty acids leads to the accumulation of triglycerides in hepatocytes; the failure to metabolize triglycerides accumulated in hepatocytes leads to fatty liver disease. This process triggers an avalanche of many factors such as an increase in the activity of cytochrome P450, an increase in the production of reactive oxygen species, lipid peroxidation, a deficiency in antioxidant defense, activation of proinflammatory cytokines and the nuclear transcription factor NF*κ*B, and an increased expression of PPAR receptors, initiating the transition from steatosis to steatohepatitis. Fibrosis and subsequent cirrhosis are the final stages in this process [60].

Our results showed an increasing superoxide dismutase activity in animals subjected to HFD. Our results agreed with the work of Sfar et al. [61]; this can be explained by the body's response to increased ROS in the prevention of alterations in cells, lipids, and proteins that are at the origin of various pathologies. These results confirm the fact that obesity is associated with oxidative stress. Cellular adaptation to this state is, therefore, the increase in the activity of SOD, which is a metalloprotein possessing enzymatic activity catalyzing the dismutation of superoxide anions into dioxygen and hydrogen peroxide [62].

*Scolymus* induced an increase in the catalase and the SOD activity, which is consistent with the work of Hermenean et al. [63]. Our results suggest that golden thistle induces an antioxidant effect, which may be linked to the richness of this plant in bioactive and antioxidant compounds, inhibiting the alterations caused by the excessive production of free radicals [64]. The extract of the *Scolymus* plant contains various flavonoids and polyphenol acids such as “catechin, rutin and tannic acid, gallic acid, pyrogallol, and chlorogenic acid,” which can contribute to antioxidant defenses in different ways by direct scavenging of free radicals and inhibition of enzymes responsible for the production of free radicals or by maintaining the integrity of mitochondrial electrons transport under stress conditions [38]. Polyphenols participate in the maintenance of an optimal redox state of the cell by activating a range of antioxidant enzymes. Polyphenols modulate the signaling pathways Nrf2/Keap1/ARE and NF*κ*B, resulting in an increase in the expression of genes encoding cytoprotective molecules. In addition, a decrease in the expression of genes modulated by NF*κ*B would reduce the production of proinflammatory cytokines [65].

Our results indicated that the level of serum erythrocyte and hepatic TBARs increases in the rats subjected to HFD, which is in agreement with the work of Feng et al. [44]. The 40% hyperfatty diet is closely related to the onset of oxidative stress and causes an increase in free radicals, which will cause the oxidation of polyunsaturated lipids, which is a free radical chain reaction process known as lipid peroxidation that gives primary products such as hydroperoxides or terminal secondary products such as malondialdehyde (MDA) [66]. In addition, the treatment with *Scolymus* decreases the level of TBARs in animals subjected to HFD. Our results are in agreement with the work carried out by Frederico et al. [67].

Prolonged consumption of HFD resulted in high levels of carbonylated proteins and advanced oxidized protein products, which is consistent with the work of Feng et al. [44]. Oxidation can affect proteins and generate advanced products of oxidized proteins, which are uremic toxins formed by the reaction of plasma proteins and, more particularly, albumin with oxidants. These oxidants are produced specifically by myeloperoxidase (MPO) secreted by activated neutrophils [68]. Any radical attack on an amino acid will cause the oxidation of certain residues resulting in the appearance of carbonyl groups in the cleavage of peptide chains and intra and interchain bityrosine bridges. Most of the damage is irreparable and can lead to major functional changes [68].

*Scolymus hispanicus* induced a decrease in AOPP and PC in the rats subjected to HFD, which is consistent with the work of Ramadan et al. [64] and Feng et al. [44]. This decrease may be due to the active components of *Scolymus* having a powerful antioxidant activity by scavenging free radicals such as hydroxyl and superoxide anion, making them inactive and stable thanks to their hydroxyl group [69].

Our results showed an increase in inflammation markers “NO and NF*κ*B” in the liver of animals subjected to a hyperfatty diet. Treatment with *Scolymus* extract decreases the levels of NF*κ*B and nitrites. These results are consistent with those in the work of Berdja et al. [25] and Smail et al. [70]. The lipophilic metabolites from *Scolymus hispanicus* L. compounds exhibited anti-inflammatory activities in vitro, manifested by inhibition of NF*κ*B p65 expression and subsequent decrease of inflammatory cytokines: IL-6, IL-1b, and TNF-*α* in PHA-stimulated with human PBMCs [71].

## 5. Conclusion

In summary, we provide proof that *Scolymus hispanicus* corrects metabolic disorders by exerting a hypoglycemic, hypolipidemic effect, improving glucose tolerance and the state of hepatic steatosis by reducing inflammation with the persistence of fibrosis and decreasing oxidative stress and inflammation in animals subjected to the hyperfatty diet.

The presence of bioactive molecules in the extract of *Scolymus* could give it anti-inflammatory and hepatoprotective antioxidant potential. Its incorporation into a dietary program or being used as a nutritional complement may be strategically effective in preventing metabolic syndrome and attenuating free radical attack, which will help prevent hepatic complications associated with a hyperfatty diet.

## Figures and Tables

**Table 1:** Total phenolic and flavonoid contents and antioxidant activity of *Scolymus hispanicus*.

Extract/standards	Total phenolic content (*µ*g GAE/ mg)	Total flavonoids (ug QE/mg)	DPPH (IC50) (*µ*g/mL)
Aqueous extract	270.321 ± 25.44	164.94 ± 9.45	0.00383
BHA	n.a.	n.a.	21.18 ± 0.12
BHT	n.a.	n.a.	12.66 ± 0.18

Each value was expressed as means ± standard deviations for triplicate experiments. n.a.: not applied. Q: quercetin; QE: quercetin equivalents; GA: gallic acid; GAE: gallic acid equivalents; BHA: butylhydroxyanisole; BHT: butylhydroxytoluene.

**Table 2:** Eight-week evolution of body weight and the plasma biochemical parameters after hyperfatty diet (40%) and normal diet feeding in rats.

	Normal diet	Hyperfat diet	ND + Sh	HFD + Sh
*Baseline*				
Body weight (g)	116 ± 4	116 ± 5 (*p* > 0.05)	118 ± 3 (*p* > 0.05)	115 ± 6 (*p* > 0.05)
Glucose (g/L)	0.94 ± 0.5	0.91 ± 0.03(*p* > 0.05)	0.92 ± 0.04 (*p* > 0.05)	0.93 ± 0.05 (*p* > 0.05)
Triglycerides (g/L)	0.45 ± 0.06	0.40 ± 0.05 (*p* > 0.05)	0.50 ± 0.09 (*p* > 0.05)	0.48 ± 0.08 (*p* > 0.05)
Total cholesterol (g/L)	0.70 ± 0.05	0.61 ± 0.11 (*p* > 0.05)	0.65 ± 0.1 (*p* > 0.05)	0.56 ± 0.06 (*p* > 0.05)
Total lipid (g/L)	2.18 ± 0.12	1.93 ± 0.24 (*p* > 0.05)	2.11 ± 0.27 (*p* > 0.05)	1.92 ± 0.09 (*p* > 0.05)
TGO (ASAT) (UI/L)	146.25 ± 13.79	141.24 ± 0.83 (*p* > 0.05)	145.08 ± 7.27 (*p* > 0.05)	134.38 ± 11.07 (*p* > 0.05)
TGP (ALAT) (UI/L)	94.9 ± 6.65	97 ± 5.48 (*p* > 0.05)	81.6 ± 13.29 (*p* > 0.05)	84.54 ± 14.47 (*p* > 0.05)
Insulin (UI)	76.2 ± 6.18	76 ± 5.24 (*p* > 0.05)	76 ± 5.55 (*p* > 0.05)	73 ± 5.83 (*p* > 0.05)

*After 8 weeks*				
Body weight (g)	134 ± 4	163 ± 8 (*p* > 0.05)	135 ± 2 (*p* > 0.05)	148 ± 8 (*p* > 0.05)
Glucose (g/L)	0.91 ± 0.04	1.17 ± 0.04^*∗∗∗∗*^	0.95 ± 0.07 (*p* > 0.05)	1.02 ± 0.11 (*p* > 0.05)
Triglycerides (g/L)	0.56 ± 0.12	2.1 ± 0.3^*∗∗∗∗*^	0.43 ± 0.07 (*p* > 0.05)	1.57 ± 0.31^*∗∗∗∗*^; (*p* > 0.05)
Total cholesterol (g/L)	0.66 ± 0.04	1.24 ± 0.03^*∗∗∗∗*^	0.6 ± 0.11 (*p* > 0.05)	1.06 ± 0.13^*∗∗∗∗*^, (*p* > 0.05)
Total lipid (g/L)	2.20 ± 0.17	5.21 ± 0.28^*∗∗∗∗*^	1.97 ± 0.33 (*p* > 0.05)	4.22 ± 0.31^*∗∗∗∗*^;^+++^
TGO (ASAT) (UI/L)	143.13 ± 14.46	220.5 ± 10.77^*∗∗∗*^	147.9 ± 6.42 (*p* > 0.05)	145.74 ± 23.07 *p* > 0.05;^+++^
TGP (ALAT) (UI/L)	99.48 ± 6.3	135.8 ± 10.15^*∗∗∗*^	97 ± 5.48 (*p* > 0.05)	104.22 ± 14.9 *p* > 0.05^;^^+++^
Insulin (UI)	80.4 ± 8.6	244.8 ± 98.42^*∗∗*^	78.8 ± 7.5 (*p* > 0.05)	124.78 ± 9.33^*∗∗∗∗*^^;+^

Effect of *Scolymus hispanicus* at 100 mg/kg for eight consecutive days. Data were expressed as mean ± standard deviation (SD) (*n* = 7). ND: normal diet; HFD: hyperfatty diet (40%) for eight weeks; ND + Sh: normal diet + *Scolymus hispanicus* extract (100 mg/kg of body weight/day during the last eight days of experimentation); HFD + Sh: hyperfatty diet treated with aqueous extract of *S. hispanicus*. *p* > 0.05 was not statistically significant. Data were expressed as mean ± standard deviation (SD) (*n* = 7). ND: normal diet; HFD: hyperfatty diet (40%) for eight weeks; ND + Sh: normal diet + *Scolymus hispanicus* extract (100 mg / kg of body weight/day during the last eight days of experimentation); HFD + Sh: hyperfatty diet treated with aqueous extract of *S. hispanicus*. *p* > 0.05 was considered not statistically significant. The symbol ^*∗*^corresponds to the comparison between HFD versus ND and HFD + SH versus ND + SH; the + symbol corresponds to the comparison between ND + SH versus NS and HFD + SH versus HFD. ^*∗∗*^*p* < 0.01; ^*∗∗∗*^*p* < 0.001; ^*∗∗∗∗*^*p* < 0.001 (HFD versus ND), ^*∗∗∗∗*^*p* < 0.001 (HFD + *Sh* versus ND + Sh), ^+++^*p* < 0.001; ^+++^*p* < 0.001 (*HFD* + *Sh* versus *HFD*).

**Table 3:** Changes of redox states in the blood (sera and erythrocytes) in rats subjected to hyperfatty diet (40% for eight weeks).

Redox states	Blood	ND	HFD	ND** **+** **Sh	HFD** **+** **Sh
Catalase (UI/mg protein)	Sera	0.17** **±** **0.01	0.06** **±** **0.004^*∗∗∗∗*^	0.35** **±** **0.02^++++^	0.29** **±** **0.06 *p* > 0.05^;++++^
	Erythrocytes	1.28** **±** **0.03	0.89** **±** **0.03^*∗∗∗∗*^	2.19** **±** **0.06^++++^	4.05** **±** **0.34^*∗∗∗*^^,++++^

TBARs (*µ*M)	Sera	65.6** **±** **4.33	95.8** **±** **2.59^*∗∗∗∗*^	67.6** **±** **2.07 (*p* > 0.05)	76.2** **±** **7.19^*∗*^^;+++^
	Erythrocytes	102.8** **±** **0.83	146.6** **±** **3.85^*∗∗∗∗*^	100.4** **±** **2.6 (*p* > 0.05)	94** **±** **4.18^*∗∗*^^;++++^

Effect of aqueous extract of *Scolymus hispanicus* (Sh) at 100 mg/kg for eight consecutive days. Data were expressed as mean** **±** **standard deviation (SD) (*n* = 7). ND*:* normal diet; HFD: hyperfatty diet (40%) for eight weeks; ND + Sh: *normal diet* + *Scolymus hispanicus* extract (100 mg/kg of body weight/day during the last eight days of experimentation), HFD + Sh: hyperfatty diet treated with aqueous extract of *S. hispanicus*. *p* > 0.05 was considered not statistically significant. The symbol ^*∗*^ corresponds to the comparison between HFD versus ND and HFD + SH versus ND + SH; the + symbol corresponds to the comparison between ND + SH versus NS and HFD + SH versus HFD. ^++++^*p* < 0.0001 (ND + Sh versus ND), ^*∗∗∗∗*^*p* < 0.001 (HFD versus *ND*), ^*∗*^*p* < 0.05; ^*∗∗∗*^*p* < 0.001, ^*∗∗∗∗*^*p* < 0.0001 (HFD + Sh versus ND + Sh), ^+++^*p* < 0.001; ^++++^*p* < 0.0001 (HFD *+* Sh *versus* HFD).

**Table 4:** Evaluation of glycogen, total lipids, and triglycerides in hepatic tissue in control and experimental groups and effect of *Scolymus hispanicus*.

Hepatic tissue	ND	HFD	ND ± Sh	HFD ± Sh
Glycogen (mg /100** **g of liver)	988 ± 87	704 ± 176^*∗∗∗∗*^	1150 ± 88^+^	1307 ± 405^*∗*^^;++++^
Total lipids (mg/100** **g of liver)	5330.83± 309.26	5803.83 ± 309.23^*∗∗∗∗*^	5137.87 ± 714.86 (*p* > 0.05)	6176.36 ± 372.81^*∗*^^;+++^
TG (mg/100** **g of liver)	906.72 ± 49.07	1751.74 ± 181.03^*∗∗∗∗*^	872.32 ± 120.83 (*p* > 0.05)	1047.36 ± 62.25^*∗*^^;++++^
Cholesterol (mg/100** **g of liver)	1769.64 ± 104.23	2804.54 ± 289.82^*∗∗∗∗*^	1706.22 ± 237.69 (*p* > 0.05)	2051.61 ± 124.30^*∗*^^;+++^

Data were expressed as mean ± standard deviation (SD) (*n* = 7). ND: normal diet; HFD: hyperfatty diet (40%) for eight weeks; ND + Sh: normal diet + *Scolymus hispanicus* extract (100 mg/kg of body weight/day during the last eight days of experimentation); HFD + Sh: hyperfatty diet treated with aqueous extract of *S. hispanicus.p* > 0.05 was not statistically significant. The symbol ^*∗*^ corresponds to the comparison between HFD versus ND and HFD + SH versus ND + SH; the + symbol corresponds to the comparison between ND + SH versus NS and HFD + SH versus HFD. ^+^*p* < 0.05 (ND + Sh versus ND), ^*∗∗∗∗*^*p* < 0.0001 (HFD versus ND), ^*∗*^*p* < 0.05 (HFD + Sh versus ND + Sh), ^+++^*p* < 0.001; ^++++^*p* < 0.0001 (HFD *+* Sh *versus* HFD).

**Table 5:** Effect of *S. hispanicus* on hyperfatty diet on redox states in liver.

Redox states	ND	HFD	ND** **+** **Sm	HFD** **+** **Sm
Catalase (UI/100** **mg of liver)	0.72 ± 0.04	0.27 ± 0.07^*∗∗∗∗*^	0.88 ± 0.07^++^	0.43 ± 0.04^*∗∗∗∗*^^;++^
SOD (UI/100 mg of liver)	1.86 ± 0.53	4.22 ± 0.66^*∗∗∗*^	2.04 ± 0.17 (*p* > 0.05)	5.95 ± 1.1^*∗∗*^^;+^
TBARs (*µ*mol/100 mg of liver)	0.43 ± 0.09	1.01 ± 0.08^*∗∗∗∗*^	0.39 ± 0.04 (*p* > 0.05)	0.53 ± 0.15 *p* > 0.05^;^^++^
Protein carbonyls (pmol/100 mg of liver)	3.87 ±0.16	6.97 ± 1.8^*∗∗*^	3.93 ± 0.12 (*p* > 0.05)	3.58 ± 0.59 *p* > 0.05^;++^
AOPP (pmol/100 mg of liver)	356.76 ± 29.01	953.63 ± 220.5^*∗∗*^	353.55 ± 29.66 (*p* > 0.05)	431.65 ± 99.96 *p* > 0.05^;++^

Data were expressed as mean ± standard deviation (SD) (*n* = 7). ND: normal diet; HFD: hyperfatty diet (40%) for eight weeks; ND + Sh: normal diet + *Scolymus hispanicus* extract (100 mg/kg of body weight/day during the last eight days of experimentation); HFD + Sh: hyperfatty diet treated with aqueous extract of *S. hispanicus*. *p* > 0.05 was not statistically significant. The symbol ^*∗*^ corresponds to the comparison between HFD versus ND and HFD + SH versus ND + SH; the + symbol corresponds to the comparison between ND + SH versus NS and HFD + SH versus HFD. ^++^*p* < 0.01 (ND + Sh versus ND), ^*∗∗*^*p* < 0.01; ^*∗∗∗*^*p* < 0.001; ^*∗∗∗∗*^*p* < 0.0001 (HFD versus ND), ^*∗∗∗*^*p* < 0.001, ^*∗∗∗∗*^*p* < 0.0001 (HFD + Sh versus ND *+* Sh), ^+^*p* < 0.05 ; ^++^*p* < 0.01; ^++++^*p* < 0.0001 (HFD + Sh *versus* HFD).

**Table 6:** Changes of inflammatory markers in the liver of the rats subjected to hyperfatty diet (40% for eight weeks).

Hepatic tissue	ND	HFD	ND** **+** **Sh	HFD** **+** **Sh
NO (Pmol/100** **mg of liver)	97** **±** **3.54	146** **±** **3.39^*∗∗∗∗*^	92** **±** **3.16^+^	112** **±** **3.16^*∗∗∗∗*^^;++++^
NF*κ* B P 35 (Pg/100** **mg of liver)	92.17** **±** **3.88	279.09** **±** **31.12^*∗∗∗∗*^	84.77** **±** **10.35 (*p* > 0.05)	101.5 ± 25.04 *p* > 0.05^;++++^

Effect of *Scolymus hispanicus* extract at 100 mg/kg for eight consecutive days. Data were expressed as mean** **±** **standard deviation (SD) (*n *** **=** **7). ND: normal diet; HFD: hyperfatty diet (40%) for eight weeks; ND** **±** **Sh: normal diet** **±**  ***Scolymus hispanicus* extract (100 mg/kg of body weight/day during the last eight days of experimentation); HFD + Sh: hyperfatty diet treated with aqueous extract of *S. hispanicus*. *p* > 0.05 was not statistically significant. The symbol ^*∗*^ corresponds to the comparison between HFD versus ND and HFD + SH versus ND + SH; the + symbol corresponds to the comparison between ND + SH versus NS and HFD + SH versus HFD. ^+^*p* < 0.05 (ND + Sh versus *ND*); ^*∗∗∗∗*^*p* < 0.0001 (*HFD* versus *ND*); ^*∗∗∗∗*^*p* < 0.0001 (*HFD* + *Sh* versus *ND* + *Sh*); ^++++^*p* < 0.0001 (*HFD* + *Sh versus HFD*).

## Data Availability

All data used to support the findings of this study are included within the article.
